# Genetic Variability in the E6, E7, and L1 Genes of Human Papillomavirus Types 16 and 18 among Women in Saudi Arabia

**DOI:** 10.3390/v15010109

**Published:** 2022-12-30

**Authors:** Madain Alsanea, Asma Alsaleh, Dalia Obeid, Faten Alhadeq, Basma Alahideb, Fatimah Alhamlan

**Affiliations:** 1 Department of Infection and Immunity, King Faisal Specialist Hospital and Research Center, Riyadh 11564, Saudi Arabia; 2 Department of Botany and Microbiology, College of Science, King Saud University, Riyadh 11564, Saudi Arabia; 3 Public Health Laboratories, Public Health Authority, Riyadh 11564, Saudi Arabia; 4 Department of Pathology and Laboratory Medicine, King Faisal Specialist Hospital and Research Centre, Riyadh 11564, Saudi Arabia; 5 College of Medicine, Alfaisal University, Riyadh 11564, Saudi Arabia

**Keywords:** HPV16, HPV18, E6 gene, E7 gene, L1 gene, cervical cancer, cancer progression, mutations, lineages, variants

## Abstract

Cervical cancer is the eighth most frequent cancer in Saudi Arabia, and most cases are associated with human papillomavirus (HPV) types 16 and 18. HPV-induced carcinogenesis may be associated with the intra-type variant, genetic mutation, or the continuous expression of viral oncogenes E6 and E7. Infection efficiency and virus antigenicity may be affected by changes in the L1 gene. Thus, this retrospective cohort study analyzed E6, E7, and L1 gene mutations in cervical specimens collected from Saudi women positive for HPV16 or HPV18 infection. HPV16 and HPV18 lineages in these specimens were predominantly from Europe. The L83V mutation in the E6 gene of HPV16 showed sufficient oncogenic potential for progression to cervical cancer. By contrast, the L28F mutation in the E7 gene of HPV16 was associated with a low risk of cervical cancer. Other specific HPV16 and HPV18 mutations were associated with an increased risk of cancer, cancer progression, viral load, and age. Four novel mutations, K53T, K53N, R365P, and K443N, were identified in the L1 gene of HPV16. These findings for HPV16 and HPV18 lineages and mutations in the E6, E7, and L1 genes among women in Saudi Arabia may inform the design and development of effective molecular diagnostic tests and vaccination strategies for the Saudi population.

## 1. Introduction

Human papillomaviruses (HPVs) are a group of small (8 kbp), non-enveloped, double-stranded DNA viruses [1,2]. Their genome can be divided into three major regions: (1) the early region (E), which contains six common open reading frames (E1, E2, E4, E5, E6, and E7) that encode nonstructural proteins; (2) the late region (L), which is divided into two open reading frames that encode one major (L1) and one minor (L2) capsid protein; and (3) the long control region, also known as the noncoding region, which does not have a protein-coding function but contains the origin of replication as well as multiple binding sites for transcription factors [1,3,4,5,6].

This group of viruses is responsible for numerous epithelial cell infections, varying from warts to cancer [7,8]. Low-risk HPV types, such as types HPV6 and HPV11, are mainly observed in genital warts, whereas high-risk HPV types, such as HPV16 and HPV18, are found in precancerous lesions and cervical cancer [7]. In 2020, cervical cancer was the fourth most common cancer among women worldwide [9]. In Saudi Arabia, cervical cancer is the eighth most frequent cancer among women between the ages of 15 and 44 years, with an estimated 358 new cases and 179 deaths occurring annually [10]. 

HPV16 and HPV18 account for 70% or more of all cervical cancer cases reported worldwide [11,12,13,14]. In Saudi Arabia, HPV16 and HPV18 were the most common types detected in 63.4% and 11.1% of cervical cancer samples, respectively, in 2011 [15]. In 2020, a 10-year retrospective analysis conducted in Saudi Arabia found that HPV16 was detected in 56.3% of cervical cancer samples, and HPV18 was detected in 7.3% of cervical cancer samples [16]. However, data assessing the HPV burden in the general population are not available [10]. 

Some HPV infections progress to high-grade cervical lesions and cancer. This means that there must be additional factors related to either the virus or the host contributing to virus persistence and progression [17,18]. Viral factors, such as virus integration, high-risk (HR)-HPV mutations, and viral load, and the presence of host-related factors, including age, marital status, number of sexual partners, number of pregnancies, and smoking, may increase the risk of virus persistence and influence the progression to cancer [19,20,21,22,23]. 

Some genetic mutations alter the biological function of the protein encoded by the mutant gene, which in turn may influence the persistence of infection, the progression of lesions to cancer, and morbidity from cervical cancer [24,25,26]. The rate of spontaneous mutagenesis is exceedingly low in normal human cells, but it dramatically increases with the expression of high-risk HPV E6/E7 proteins [5]. When oncogenes E6 and E7 act cooperatively, they interact with and inhibit the activities of tumor suppressors to enable malignant cell transformation [8,27]. The L1 gene, which plays an important role in the initial interaction of the capsid with host cells and mediates the integration into the nucleus [6,24,28], can be used to design HPV detection primers for clinical use [29,30]. The identification, screening, and analyses of various genomic regions of HR-HPV genotypes, such as HPV16 and HPV18, are important for developing HPV diagnostics, vaccines, and other therapeutic approaches to managing HPV-induced carcinogenesis [24,26,31,32].

Because the accurate detection and typing of HPVs are essential in screening and disease management protocols, the present study aimed to investigate the genetic variability in the E6, E7, and L1 genes of HPV16 and HPV18 in HPV-positive specimens isolated from Saudi female patients. Our study also aimed to assess HPV16 and HPV18 genetic mutations and the risk of cervical cancer in these specimens. By understanding the genetic variability in our population, we can design better screening programs and improved assays. 

## 2. Materials and Methods

### 2.1. Ethics Statement

This retrospective cohort study was approved by the Research Advisory Council (Ethics Committee) at King Faisal Specialist Hospital and Research Centre (approval number: RAC 1005-033) with waived consent because the cervical biopsies were archived, and the collected patient data were coded and thus deidentified.

### 2.2. Study Population

In total, 1460 archived cervical specimens or biopsy specimens obtained from women attending Family Medicine and OB/GYN clinics at King Faisal Specialist Hospital and Research Centre in Riyadh were included in this study. All specimens were collected between 2017 and 2019. Clinical and sociodemographic data, as well as the viral load data of the specimens, were obtained from previous studies [23,33,34]. Participant inclusion criteria for the present study were (1) being a Saudi woman (2) who was married, divorced, or widowed, (3) having definite histological or cytological results for collected cervical specimens, and (4) having an HPV genotype test result positive for HPV16 or HPV18 infection. Abnormal cytological stages were identified using the Bethesda classification system [35]. A post-inclusion criterion was that the total sample size was 60. 

### 2.3. Genomic DNA Extraction and Quality Control

For specimens obtained through Pap testing, DNA was extracted by using a Gentra Puregene Cell Kit according to the manufacturer’s instructions (Qiagen, Hilden, Germany). For formalin-fixed paraffin-embedded cervical specimens, DNA was extracted using a QIAamp DNA FFPE Tissue Kit by following the manufacturer’s instructions (Qiagen, Valencia, CA, USA). The β-globin housekeeping gene was tested to ensure the quality of the genomic DNA. The housekeeping gene primers used are given in Table 1.

The PCR cycling parameters are given in Table 2. The amplified products were visualized using 1.5% agarose gels stained with Green View™ Plus that had been electrophoresed for 90 min at 120 volts per meter. A 100-base-pair (bp) DNA ladder was used to identify the product size. The Gel Doc™ EZ imaging system from Bio-Rad was used to capture images.

### 2.4. HPV Detection and Genotyping

Well-established sets of nested PCR primers were used to detect the L1 region in the HPV genome, including the MY09/MY11 primer set, which targets a 450 bp conserved sequence, and the GP5+/GP6+ primer set, which targets a 150 bp sequence within the 450 bp product. For positive controls, SiHa and HeLa cell lines were used for HPV16 and HPV18, respectively. HPV genotyping was performed by using two assays: a reverse line blotting hybridization assay that used 23 oligonucleotide probes and a reverse dot blot (GenoFlow) HPV array test (Diagcor Bioscience Incorporation Limited; Hong Kong), with the ability to genotype 33 HPV types. All typing assays have been described previously [36]. Viral loads for HPV16 and HPV18 were assessed using in-house relative assays (TaqMan), which have also been described previously [37]. Both assays were performed using the cycling conditions given in Table 2 with the 7500 Fast Real-Time PCR system and software (Applied Biosystems, Foster City, CA, USA).

### 2.5. Amplification of Target Genes

In-house target-specific primers for HPV16 E6, E7, and L1 genes and for HPV18 E6, E7, and L1 genes were designed using Primer3 version 4.1.0 (https://primer3.ut.ee/ (accessed on 3 February 2021). The primers were designed referring to the HPV16 (NC_001526) and HPV18 (NC_001357.1) prototypes published in GenBank (National Center for Biotechnology Information; NCBI) [30]. All primers are shown in Table 3 and Table 4. 

For annealing temperature optimization, gradient PCR was performed. The 23 μL reaction solutions contained 12.5 μL of GoTaq^®^ Green Master Mix (Promega, Madison, WI USA,), 9.5 μL of UltraPure DNase/RNase–free water, 0.5 μL of forward primer, and 0.5 μL of reverse primer. The primer concentration was 95 pmol (5 μL of primer added to 95 μL of 1X TE buffer) and included 2 μL of the DNA template. SiHa and HeLa cell lines were used for HPV16 and HPV18, respectively. For the negative control, UltraPure DNase/RNase–free water was used in this optimization. For the HPV16 primers, the temperature for touchdown PCR ranged from 64 °C to 53 °C, and the HPV18 range was from 61 °C to 56 °C. Cycling parameters for both HPV16 and HPV18 primers are given in Table 2. The bands resulting from the amplified products were compared visually after gel electrophoresis. 

### 2.6. Sequencing and Sequence Analysis

The PCR products were sequenced at the Sequencing Core Facility, Department of Genetics, King Faisal Specialist Hospital and Research Centre, using an ABI3730XL DNA Analyzer (Applied Biosystems, Foster City, CA, USA). Sanger shotgun sequencing was performed for the L1 gene. HPV16 and HPV18 L1 genes were fragmented into three pieces. The HPV16 fragments were 660 bp, 669 bp, and 864 bp, and the HPV18 fragments were 700 bp, 698 bp, and 696 bp. DNASTAR Lasergene 15.0 package SeqMan Pro, version 15, was used for sequence analyses and the assembly of the L1 gene contigs. Sequences were then aligned against the prototype references NC_001526 for HPV16 and NC_001357.1 for HPV18 to detect variants by using ClustalW and MegAlign Pro, version 15.

To determine the amino acid variants, all sequences were translated into amino acids using EditSeq, version 15, and realigned by MUSCLE to the following reference protein sequences: for HPV16, E6 (accession No. NP_041325.1), E7 (accession No. NP_041326.1), and L1 (accession No. NP_041332.2); for HPV18, E6 (accession No. NP_040310.1), E7 (accession No. NP_040311.1), and L1 (accession No. NC_001357.1). Both nucleotide and amino acid variant positions were revised and confirmed by using SeqBuilder Pro, version 15, and by comparing them with published variants.

### 2.7. Phylogenetic Tree Construction

Phylogenetic trees were constructed using IQ-TREE (http://iqtree.cibiv.univie.ac.at (accessed on 3 March 2021) [38] based on the E6 and L1 genes. Maximum likelihood values were calculated with 1000 replicates to test the robustness of the major phylogenetic group. FigTree 1.4 was used to view and edit the phylogenetic trees [39]. For the construction of the HPV16 and HPV18 phylogenetic trees, sublineage references were obtained from the NCBI GenBank database and the PapillomaVirus Episteme database, as shown in Table 5 [39,40].

### 2.8. Statistical Analysis

Data were analyzed using Statistical Analytical Software (SAS), version 9.4 (SAS Institute Inc.; Cary, NC, USA), and GraphPad, version 8 (San Diego, CA, USA). *T*-tests or Wilcoxon rank-sum tests were used to assess differences in numerical values between two groups. For analyses of multiple factors, analysis of variance (ANOVA) or Kruskal–Wallis tests were used, as appropriate. Chi-square tests for associations were conducted to evaluate the distribution of categorical variables. Odds ratios (ORs) were used to evaluate cancer risk by variant. Descriptive analyses were conducted to assess the distribution of cases by group with time and were visualized using bar graphs and scatter plots. A 2-sided *p*-value of alpha < 0.05 was considered statistically significant.

## 3. Results

### 3.1. Study Population

**Figure 1:**
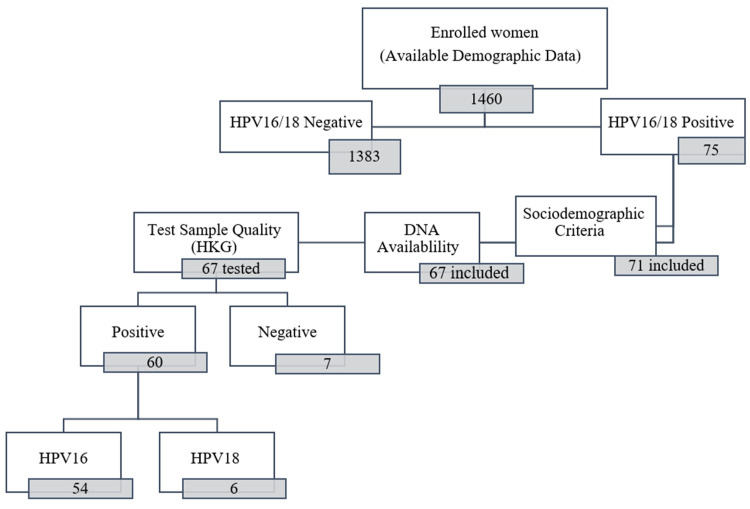
Schematic of the final study sample population after application of inclusion and exclusion criteria. Numerals in boxes represent the number of specimens. HKG indicates the housekeeping gene assay.

Tests assessing a housekeeping gene were performed on 67 archived specimens that had passed our initial inclusion criteria. We found 60 (89.6%) specimens that were positive for the housekeeping gene, and we excluded 7 (10.4%) specimens that were negative for the gene. Of 60 specimens included in the ensuing analyses, 54 specimens were positive for the presence of HPV16, and 6 specimens were HPV18-positive (Figure 1).

In total, 70.3% of the HPV16-positive specimens and 100% of the HPV18-positive specimens were obtained from married women. The mean (SD) age of the women providing the HPV16-positive specimens was 53.9 (12.9) years, and the mean (SD) age of the women providing the HPV18-positive specimens was 48 (8.9) years (Table 6). Approximately 81% of the HPV16-positive specimens were collected from women who were diagnosed as having cervical cancer, whereas all of the HPV18-positive specimens were collected from women who were diagnosed as having cervical cancer. The histology and cytology results of all cervical tissues collected for this study are given in Table 6.

### 3.2. Sequence Analyses

For specimens that were HPV16-positive (N = 54), 72 nucleic acid variants were detected: 19 were located in the E6 gene, 11 were in the E7 gene, and 42 were in the L1 gene. Among these 72 variants, 30 resulted in amino acid mutations, with 12 of these amino acid mutations detected in the E6 gene, 2 in the E7 gene, and 16 in the L1 gene. A summary of the frequencies and percentages of the detected variants is given in the Supplementary Materials Table S1. 

In 6 sequences that were obtained from HPV18-positive specimens, 20 nucleic acid mutations were detected, 4 in the E6 gene, 4 in the E7 gene, and 12 in the L1 gene. Among these 20 nucleic acid mutations, 8 resulted in amino acid mutations, 2 in the E7 gene and 6 in the L1 gene. A summary of the frequencies and percentages of the detected variants is given in the Supplementary Materials Table S1.

**Figure 2:**
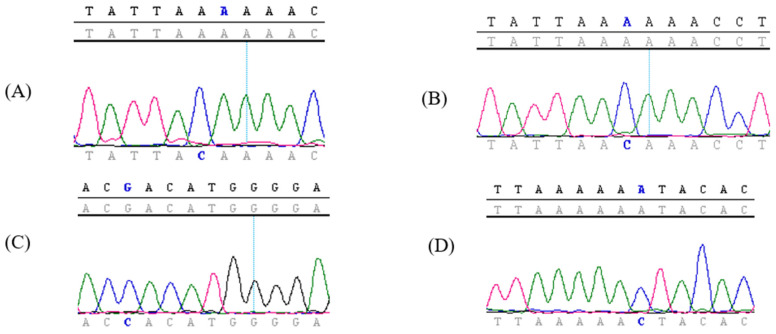
Novel mutations detected in the study population. (**A**) In specimen 219, A5796C led to changing lysine in position 53 to threonine (K53T). (**B**) In specimen 184, A5797C led to changing lysine in position 53 to asparagine (K53N). (**C**) In specimen 163, G6732C led to changing arginine in position 365 to proline (R365P). (**D**) In sample 136, A6967C led to changing lysine in position 443 to asparagine (K443N).

The HPV16 L1 gene had four nonsynonymous novel mutations: K53T, K53N, R365P, and K443N. The chromatograms are shown in Figure 2.

### 3.3. Association of HPV16 Variants with Cervical Cancer Risk

We evaluated the risk of cervical cancer associated with the various HPV16 variants, but not the HPV18 variants because all of the HPV18-positive specimens were collected from women who had already received a diagnosis of cervical cancer. Using Fisher’s exact test, we found a significant excess risk of cervical cancer associated with three HPV16 variants, one of which was observed in each of the studied genomic regions. In the E6 gene, the highest increased risk of cervical cancer was for the L83V amino acid mutation (OR: 10.1; 95% confidence interval (CI): 1.1–89.4). In the E7 gene, the L28F mutation was associated with a significantly lower risk of cervical cancer (OR: 0.14; 95% CI: 0.07–0.27). In the L1 gene, the highest significant risk was for the variant with the T266A amino acid change (OR: 4.5; 95% CI: 0.92–21.9). Thus, the two main amino acid mutations that showed an increased risk of cervical cancer associated with HPV16 infection were L84V and T266A, whereas the L28F mutation was associated with a lower risk. All tables reporting the ORs and statistical tests are shown in the Supplementary Materials Tables S2–S4. 

### 3.4. Association of Variants with Cervical Dysplasia Evaluated by Histological and Cytological Grades

We used chi-squared tests to assess the associations of the HPV16 and HPV18 variants with the histological and cytological grades of the cervical specimens. The summary of the analysis is shown in the Supplementary Materials Tables S5–S8. For the HPV16 E6 variants, we found no association between any variant and the histological grade. A significant association with the cytological grade was detected only for the R10I mutation (χ^2^ = 6.7, *p* = 0.048). For the HPV16 E7 gene variants, no association was found between the cytological grade and gene mutation. However, a significant association was detected between the histological grade and the L28F (χ^2^ = 35, *p* < 0.0001) and T795C (χ^2^ = 10.9, *p* = 0.027) mutations. For both E6 and E7 gene mutations, there was no significant association between the gene variant and squamous cell carcinoma (SCC) grade. For HPV16 L1 gene variants, a significant association with the cytological grade was detected only for the V194I mutation (χ^2^ = 13.7, *p* = 0.001). However, significant associations were detected between the histological grade and the T266A (χ^2^ = 11.1, *p* = 0.025), G6994A (χ^2^ = 10.9, *p* = 0.028) and L474F (χ^2^ = 10.9, *p* = 0.028) mutations. Three mutations were significantly associated with the SCC grade: T353P (χ^2^ = 7.8, *p* = 0.02), G6721A (χ^2^ = 7.8, *p* = 0.02), and C6854T (χ^2^ = 7.8, *p* = 0.02).

Due to the sample size, detected mutations in the E6, E7, and L1 regions of the HPV18 variants were not evaluated by the histological, cytological, or SCC grade. 

### 3.5. Viral Load by HPV16 or HPV18 Variant

We used Wilcoxon rank-sum tests to evaluate whether specific variants were associated with the viral load. For the HPV16 E6 gene, five mutations (G145T, Q14H, T286A, A289G, and H78Y) were associated with higher viral loads (Table 7). For the HPV16 E7 gene, two mutations, T789C and T795G, were significantly associated with higher viral loads. For the HPV16 L1 gene, 12 mutations (G5698A, H76Y, T5911C, T266A, T353P, G6721A, C6854T, C6865T, C6970T, G6994A, and L474F) were significantly associated with higher viral loads.

For HPV18, due to the small sample size, we could not perform the statistical test.

### 3.6. Phylogenetic Trees

**Figure 3:**
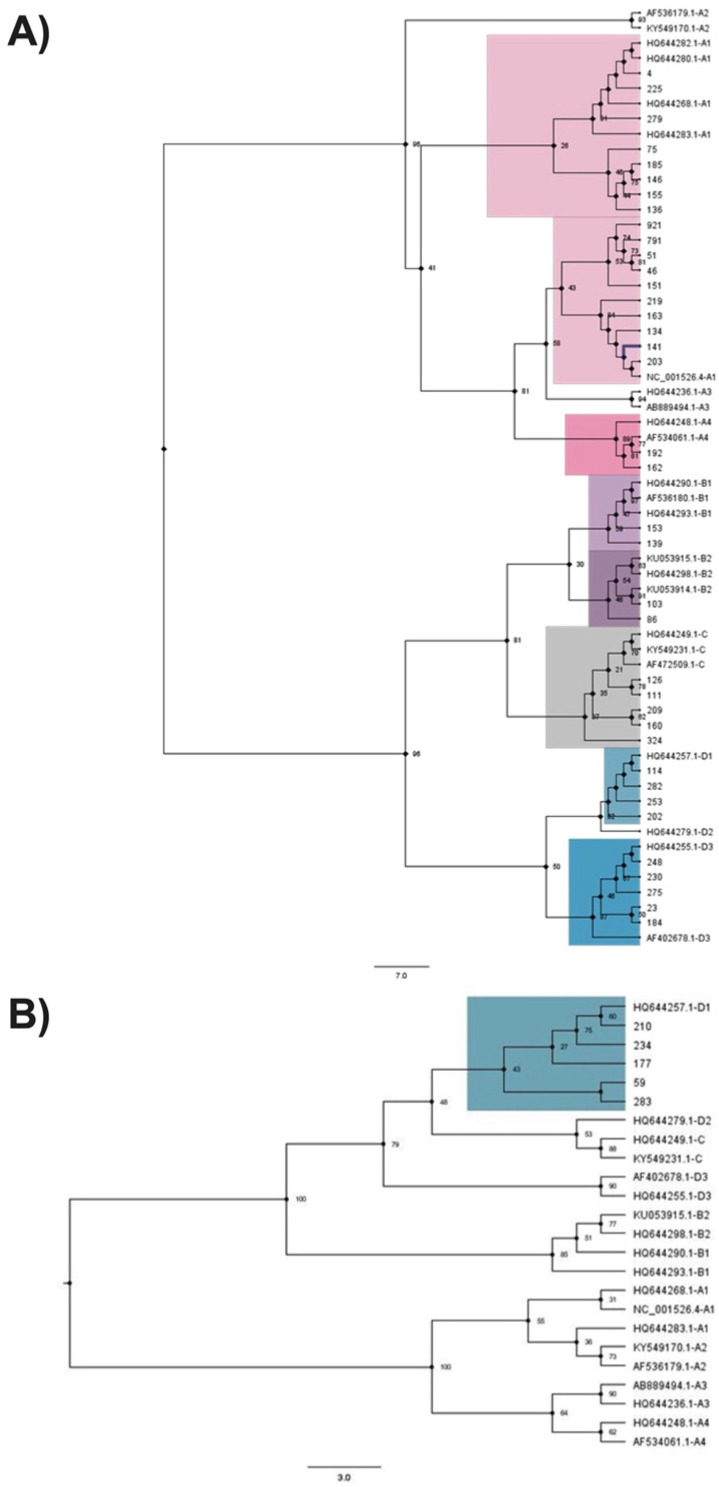
HPV16 phylogenetic trees. (**A**) Maximum likelihood analysis of the E6 nucleotide sequence. Specimens that did not cluster to any reference sequence were excluded. (**B**) Excluded specimens with complete L1 genes were reanalyzed by using maximum likelihood of the L1 nucleotide sequence. Numbers on the nodes indicate bootstrap values.

For HPV16, 43 of 54 (79.6%) specimens were successfully classified, 38 specimens based on the E6 gene and 5 based on the complete L1 gene. The results of the lineage analysis showed that 46.5% of the specimens belonged to lineage A, 9.3% belonged to lineage B, 11.6% belonged to lineage C, and 32.6% belonged to lineage D. For lineage A, 41.9% of the specimens were classified as A1, which is a European lineage, and 4.7% were classified as A4, which is an Asian lineage. For lineage B, 4.7% of the specimens were classified as both B1 and B2, both of which are African lineages. For lineage C, 11.6% belonged to an African lineage. For lineage D, 20.9% were classified as D1, which is a North American lineage, and 11.6% were classified as D3, an Asian American sublineage. The phylogenetic trees are illustrated in Figure 3. No specimens belonging to A2, A3, A5, or D2 sublineages were detected. 

**Figure 4:**
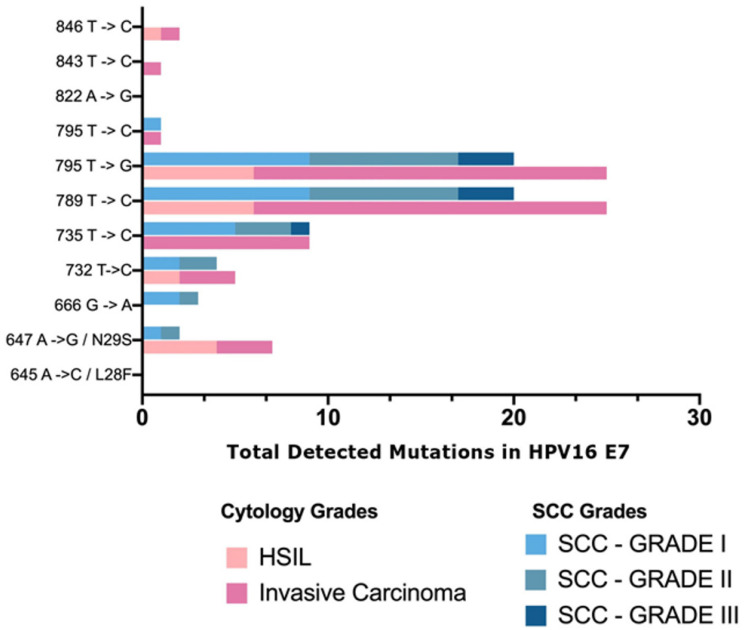
HPV18 phylogenetic tree. Maximum likelihood analysis of the E6 nucleotide sequence. Numbers on the nodes indicate bootstrap values. HSIL represents high-grade squamous intraepithelial lesion; SCC, squamous cell carcinoma.

For HPV18, 5 of 6 (83.3%) specimens were successfully classified. Only lineage A was detected, with 60% belonging to the A4 sublineage and 40% belonging to the A5 sublineage, both of which are European lineages. The phylogenetic tree is shown in Figure 4.

### 3.7. Association of Lineage with Patient Demographic Characteristics

There was no significant association between patient demographic variables and HPV16 lineages or HPV18 lineages.

## 4. Discussion

The role of HR-HPV intra-type variants and genetic mutations in the development of malignant neoplasms is still under investigation, and the results of many studies assessing the clinical and prognostic significance of HPV detection in cervical cancer screening are contentious [20,26]. E6 and E7 gene mutations may influence malignant transformation processes, including cell cycle arrest and the absence of apoptosis caused by E6 and hyperproliferation caused by E7 [12,20,26,41,42]. L1 gene mutations may change the host’s cellular immune response and may play a role in vaccine effectiveness because the L1 region is typically used to generate vaccines [11,12,20]. Therefore, identifying and understanding HR-HPV genetic mutations are essential for studying molecular pathogenesis and for developing molecular diagnostics, vaccines, and other therapeutic approaches aimed at controlling or eliminating virus-induced diseases [20,26]. The present study was, to our knowledge, the first to investigate mutations in the E6, E7, and L1 genes among Saudi women who tested positive for HPV16 or HPV18 infection. 

We found 12 nonsynonymous mutations in the HPV16 E6 gene: R10I, Q14D, Q14H, H24N, D25Y, D25E, I27R, V31A, A61G, D64E, H78Y, and L83V. Previous studies have demonstrated that R10I and Q14D mutations may affect p53 affinity and significantly alter its degradation rate [19,43,44]. D25E also plays a major role in p53 degradation [45,46]. Our results indicated that R10I was the only mutation that was significantly associated with the cytological grade (χ^2^ = 6.7, *p* = 0.048). A recent study evaluating E6 structural changes using an in silico approach found that the Q14H mutation is located at the binding interface of the E6-p53 core and thus may play an important role in p53 binding. Those authors also suggested that an I27R mutation may lead to changes in molecular interactions [47]. Although H24N, D25Y, V31A, A61G, and D64E mutations have been detected in previous studies, their structural and functional effects require further investigation [43,48,49,50,51]. 

The mutations most frequently detected in our study were H78Y and L83V, with a rate of 57.4% each. There are fewer studies assessing the role of H78Y compared with those for L83V. It has been suggested that the region where amino acid 83 is located could be responsible for the greater oncogenic potential of HR-HPVs [52]. The authors of that study conducted functional analyses of the mutations found in 10 premalignant and malignant tissues. The biochemical activities of these mutations were evaluated using both in vivo and in vitro assays. Their comparison of the L83V mutation with the E6 gene prototype indicated that L83V displayed moderate to high activities in most functional assays, more efficient binding to the E6-binding protein, and more efficient degradation of the apoptosis regulator Bax compared with the other variants [19]. These results could explain our finding of high odds for detecting the L83V amino acid mutation in cervical cancer specimens (OR: 10.1; 95% CI: 1.1–89.4).

The genetic basis of HPV carcinogenicity is geographically different among variants and mutations [53,54,55]. It is well known that both L83V and D25E mutations in the E6 gene are associated with an elevated risk of cervical carcinoma [43]. However, in the present study, the excess risk associated with cervical cancer and HPV mutation was significant only for the L83V mutation. Our results are similar to those of a hospital-based study that was conducted in Korea and reported a significant association of L83V with cervical cancer [44]. By contrast, previous studies that were performed in China did not find an association between L83V and cervical cancer [40,56]. These inconsistent results assessing the E6-L83V role in the development of cervical cancer may be explained in part by (1) the geographic origins or races of the patients, (2) the small number of cases investigated, and (3) the differences in experimental design [26,38,42]. More studies investigating the functional implications of the L83V mutation are required [57].

The L28F and N29S mutations were the only nonsynonymous E7 mutations detected in the present study, and the N29S mutation was the most prevalent mutation detected. Consistent with our findings, various studies evaluating E7 mutations have reported a higher prevalence of N29S compared with other mutations [44,54,56]. Previous studies have also reported that the E7 protein linked to cervical cancer is more conserved than the E6 protein [43]. In our study, the number of E6 mutations was higher than the number of E7 mutations by 113%. Moreover, the E7 mutation L28F was associated with a significantly lower risk of cervical cancer. It is known that L28F affects DNA synthesis, pRb binding, and nuclear localization signals. Considering these known functional implications, the association between L28F and the decreased risk of both cervical dysplasia and invasive cancer should be further investigated [44]. 

Mutations that occur in surface loops BC, DE, EF, FG, and HI or in sites between them could affect virus infection efficiency or alter virus antigenicity [11,20,58]. A Dutch study of genetic variation in the L1 proteins of HPV16 and HPV18 among young adolescents found that 78% of L1 amino acid mutations were located in the encoding region of the alpha helix, beta sheets, surface loops, or connecting loops [58,59,60]. Of the 16 amino acid mutations detected in the present study (K53N, K53T, H76Y, T176N, N181S, N181T, V194I, T266A, S282P, S351A, T353P, R365P, T389S, S396P, K443N, and L474F), 75% were located in the surface loops and in the encoding regions of the alpha helix and beta sheet. Structural analyses of the L1 protein in previous studies have shown that T176C, N181S, N181T, and V194I are located in the EF surface loop, T266A and S282P are in the FG surface loop, and S351A and T353P are in the HI surface loop. T389S, S396P, and L474F are in the alpha-helix encoding region, whereas H76Y is located in the region that encodes the beta sheet [57,58,59,60].

The most frequently detected mutations in our specimens were T176N and H76Y. In a previous study, pseudovirion (PsV) constructs of 39 L1 variants were generated to analyze the effectiveness of these variants [60]. Owing to the absence of any detectible proteins or PsV particles when investigated under the electron microscope, T176N presumably reduced the expression level or stability of L1 or affected PsV assembly. A recent study reported that T176N was a part of linear B-cell epitope regions, whereas H76Y and T266A were part of known T-cell epitopes. In our study, T266A was associated with the highest risk of cervical cancer (OR: 4.5; 95% CI: 0.92–21.9). A previous study using the Site-Directed Mutator tool to predict the effects of variants on protein stability found that T266A may result in the loss of two hydrogen bonds with phenylalanine in position 360 (F360) and lysine in the next position (K361) of the adjacent chain [61].

Previous studies have reported that HPV16 variants and mutations associated with the occurrence of cervical cancer may differ by geographic region or ethnicity [39,44,47,55,62]. One study in India found that the N181T/I, T353P, and T389S mutations are associated with the histological grade [61]. We found that the presence of V194I was significantly associated with the histological grade, whereas the presence of T266A, L474F, and G6994A was significantly associated with the cytological grade. Thus, further investigations assessing the oncogenicity of variants/mutations in larger populations are needed that take into consideration geography and ethnicity effects [44].

We identified four amino acid mutations of the HPV16 L1 gene: K53T, K53N, R365P, and K443N. To the best of our knowledge, none of these four mutations have been reported. A recent study in India reported a mutation in position 443 of this gene for the first time in Indian isolates. However, in that study, in HPV-18, lysine in position 443 was replaced by glutamine, whereas in our study, the replacement was by asparagine, which has not been previously reported. Those authors hypothesized that lysine in that position may stabilize the structure of L1 [61]. 

No amino acid mutations were detected in the E6 gene. By contrast, there were two nonsynonymous mutations in the E7 gene, L8V and M61L, with L8V being more prevalent. Previous studies have reported L8V and M61L mutations in the E7 gene; however, the biological role of these mutations will require additional investigation [43,63]. In general, genetic studies assessing HPV18 are insufficient, making comparisons difficult [64].

Viral antigenicity and infection efficiency may be affected by amino acid mutations in the L1 gene [11]. In the present study, there were six amino acid mutations: R25Q, L64M, P91R, T149N, P344R, and P399R. All of these mutations have been previously reported, although insufficient studies are available exploring their roles and effects [11,24,42,65]. The amino acid mutations most frequently detected in the present study were R25Q, P91R, and T149N. The authors of a previous study suggested that R25Q and P91R may affect immune responses to the HPV18 capsid protein [24]. Amino acid mutations at position 149 have been detected in many HPV genotypes, and these mutations may decrease the probability of other mutation effects on L1 gene function [65]. 

We also found that 19 mutations of HPV16 (5 in E6, 2 in E7, and 12 in L1) were significantly associated with higher viral loads. By contrast, none of the HPV18 mutations were significantly associated with higher viral loads, but this finding may be due to the small number of specimens assessed in the present study. A previous study investigated the same specimens we used in our study but categorized cytological grades as normal and abnormal. Their logistic regression model showed a significant association between a higher risk of cervical abnormalities and high viral loads for both HPV16 and HPV18 [33]. We used three categories of cytological grades: negative for intraepithelial lesions, high-grade squamous intraepithelial lesions, and invasive carcinoma. Consistent with the results of that previous study, we found that higher viral loads were mainly observed in invasive carcinoma, with lower viral loads detected mainly in cases negative for intraepithelial lesions and cases with high-grade squamous intraepithelial lesions. However, a cross-sectional study conducted in China reported that the HPV16 viral load increased significantly with the increased invasiveness of the cervical lesion grade and was predominant in cervical cancer, whereas the HPV18 viral load showed no significant increase, although it was slightly higher in cervical cancer cases [37]. Another cross-sectional study performed in India found no association between the HPV18 viral load and cervical lesion grade but did detect such an association for the HPV16 viral load [66]. Controlling the factors that may affect the results, such as the method of virus detection and the tissue classification system, are required to be similar to enable an accurate comparison [37]. Further studies investigating the associations between mutations, viral loads, and cervical lesion grades are needed in Saudi Arabia, as well as globally, because the studies in this region are limited. 

In Southeast Asia, Asian sublineages are the most prevalent variants, whereas European sublineages predominate in all other geographic regions except Africa [54]. According to the phylogenetic trees generated from our study, for HPV16 and HPV18, most of the sequences were clustered within the European sublineages.

A study by Sait et al. conducted in Saudi Arabia based on sequence analyses of the LI gene reported that 62.5% of HPV16 specimens belonged to lineage A, 50% of which were from European sublineages, and no specimens belonged to sublineages A3, A5, B2, D1, or D3 [31]. For HPV18, 100% of the specimens belonged to lineage A, with 71.4% of them from European sublineages, and no specimens belonged to sublineage A2 or lineages B, C, or D. Their results are consistent with ours, although we constructed phylogenetic trees based on the E6 and L1 genes separately. The most frequently detected lineage in our HPV16 and HPV18 specimens was lineage A, which represented 46% and 100% of the population, respectively. For HPV16, 41.9% of the specimens belonged to European sublineages, and no specimens belonged to the A3, A5, or D2 sublineages. For HPV18, all specimens belonged to European sublineages, and no specimens belonged to the A1 or A2 sublineages or to the B, C, or D lineages. 

The distribution of the mutations in the HPV16 and HPV18 lineages appeared to be geographically related. The L83V mutation detected in the European variant of the HPV16 E6 gene was the most prevalent in Central and South America (52%) and Europe (44%). However, the D25E mutation in the Asian variant was the most prevalent in Southeast Asia (26%), in contrast to Europe (2%), Central and South America (0%), and Africa (0%) [55]. In our study, the prevalence of L83V was 57.4%, and the prevalence of D25E was 4.3%.

In addition to this potential geographic effect, the distribution of the mutations appeared to be associated with the race of the women living in the same geographical region. Of 1025 women who were included in a race-related variant distribution study [67,68,69,70,71,72], 70% were White, 24.5% were African American, 3.5% were Asian/Pacific Islander, and 1.8% were American Indian/Alaskan living in the United States. The study found that 162 African American women were positive for HPV16, of which 43 isolates (26.5%) belonged to the African variant, whereas for 584 White women who were infected with HPV16, 25 isolates (4.3%) belonged to this variant. The statistical analysis of the distribution of HPV16 variants among women who were African American indicated a significant difference from that observed among White women (*p* < 0.001) and among American Indian/Alaskan women or among Asian/Pacific Islander women (*p* < 0.001). For HPV18 specimens, the African variant was predominant in African American women. The difference in the overall distribution of HPV18 variants between African American and White women was statistically significant (*p* < 0.001) [72]. 

Our study was conducted in Riyadh, whereas the study by Sait et al. [31] was conducted in Jeddah, where the ethnic diversity is known to be greater than that in Riyadh. This diversity may be another reason, in addition to the targeted genes and different protocols, for the reported differences in some sublineage distributions. However, the conclusions from their study and ours—that the most prevalent HPV16 and HPV18 lineages in Saudi Arabia were European—are consistent. 

A major challenge for preserving genetic resources is the high-quality long-term storage of DNA samples [68]. The samples enrolled in this study were collected between 2017 and 2019 and had been used in previously published studies. Multiple repetitive freeze–thawing cycles may affect DNA quality. When we used housekeeping gene assays to assess the DNA quality, we indeed found seven specimens that were negative for the gene and were thus excluded from the present study. In addition, 4 specimen vials were empty, and 12 specimens had insufficient DNA for use in sequencing all of the genes of interest.

The gold-standard technology for verifying PCR results and analyzing nucleic acid sequences is Sanger sequencing [68,69]. The automated technology can support the analysis of nucleic acid sequences up to 800–1000 bp [68]. In the present study, we used an automated DNA analyzer to sequence the targeted genes. All of the nucleic acid sequences ranged from 512 bp to 966 bp. However, the low quality of the first 15–40 bp of the sequences was a major limitation of this approach. These unreadable areas are known to be primer binding sites and they often contain indels. Thus, areas must be trimmed, with only high-quality target nucleic acid sequences used for sequence assembly [68]. Therefore, in-house primers were designed to flank the regions of interest of ≥15 bp. Another major limitation in our study is our use of genomic DNA from formalin-fixed paraffin-embedded specimens, which partially degraded; also, a high number of PCR cycles were performed, which could cause artifacts. However, we have sequenced each sample two times to overcome this limitation. 

Shotgun sequencing is a well-established approach [69]. In this technique, DNA is typically randomly fragmented into smaller pieces, sequenced, and assembled as a contig [69,70]. The contig is an assemblage of overlapping sequence reads [71]. However, in our study, the L1 genes of both HPV16 and HPV18 were not fragmented randomly. We used target-specific primer sets to fragment the L1 genes by binding to specific regions in three fragments. The sequence reads of the fragments were overlapped to eliminate the possibility of having unreadable areas and to guarantee the whole gene was sequenced. However, we were unable to obtain all three fragments of the L1 gene from all specimens. Unlike the E6 and E7 genes, the L1 gene can be lost during HPV genome integration during the progression from a low-grade lesion to cancer [36,72]. A previous study compared the performance of MY09/11 primers designed to target the conserved region of the L1 gene with type-specific primers, mainly directed at E6 and E7 genes, for HPV detection. The study found that the MY09/11 primers failed to detect 409 (83.3%) of the 491 HR-HPV–positive specimens that were detected with the type-specific primers. The 409 false-negative specimens were followed up, and it was found that 104 (25.4%) were classified as CIN2+ (moderate dysplasia or higher) [36]. In our study, 82.9% of the HPV16 specimens and 60% of the HPV18 specimens were classified as CIN3+ (severe dysplasia or higher), which may explain our inability to obtain the complete L1 gene from all specimens.

In conclusion, this retrospective cohort study contributes to the HPV epidemiology knowledge for a Saudi population. We provide data on the HPV16 and HPV18 lineages and on mutations in the E6, E7, and L1 genes. Our findings confirmed the predominance of the European lineage in HPV16 and HPV18 specimens in Saudi Arabia. We observed that the number of E6 mutations was 113% higher than E7 mutations in HPV16. We also provided evidence that the L83V mutation in the E6 gene of HPV16 in this Saudi population carried sufficient oncogenic potential to be responsible for the progression to cervical cancer. By contrast, the L28F mutation in the E7 gene of HPV16 was associated with a significantly low risk of cervical cancer. In addition, specific mutations in HPV16 and HPV18 were associated with the risk of cancer, cancer progression, viral load, and age, suggesting that these mutations represented increased oncogenic activity. We also identified four novel mutations, K53T, K53N, R365P, and K443N, in the L1 gene of HPV16. Although this study offers data essential for informing the design and development of effective molecular diagnostic tests and vaccination strategies for the Saudi population, further studies assessing the associations between mutations, viral loads, and cervical lesion grades are needed in Saudi Arabia as well as globally.

## Figures and Tables

**Table 1:** Housekeeping gene primers.

Primer	Sequence	Length, Bases	Product Size, Base Pairs	Melting Temperature
GH20	CAACTTCATCCACGTTCACC	20	248	58 °C
PC04	GAAGAGCCAAGGACAGGTAC	20		

**Table 2:** PCR cycling parameters.

Step	Temperature, °C	Time	Cycle
Initial Denaturation	95	5 min	1
Denaturation	95	30 s	45
Annealing	58 *	30 s	
Extension	72	30 s	
Final Extension	72	5 min	1
	4	∞	

* For HPV16 primers, the touchdown PCR ranged from 64 °C to 53 °C, whereas for HPV18, the range was from 61 °C to 56 °C.

**Table 3:** In-house target-specific primers for HPV16 E6 and E7 and the multiple primer sets for L1.

Primer		Length, Bases	Product Size, bp	Tm, °C	Gene Location (Sequence Length)
HPV16-E6 (F)	ACCGGTTAGTATAAAAGCAGACA	23	700	57.59	56–755 (699)
HPV16-E6 (R)	AGCGTAGAGTCACACTTGCA	20		59.33	
Sequence		Length, Bases	Product Size, bp	Tm, °C	Gene Location
HPV16-E7 (F)	TGTCAAAAGCCACTRTGTCC	20	553	59.73	419–972 (553)
HPV16-E7 (R)	GTCATYTGATAYRGCATCCCCTGT	24		59.83	
Sequence		Length, Bases	Product Size, bp	Tm, °C	Gene Location
HPV16-L1 fragment 1 (F)	AACTGACCAARCTCCTTCATT	21	661	57.48	5504–6164 (660)
HPV16-L1 fragment 1 (R)	GATCCTTTGCCCCAGTGTTC	20		58.82	
Sequence		Length, Bases	Product Size, bp	Tm, °C	Gene Location
HPV16-L1 fragment 2 (F)	TGGATGACACMGAAAATGCTAGT	23	678	58.09	6023–6692 (669)
HPV16-L1 fragment 2 (R)	CTGAAGTWGATATGGCAGCACA	22		57.94	
Sequence		Length, Bases	Product Size, bp	Tm, °C	Gene Location
HPV16-L1 fragment 3 (F)	GAACCATATGGCGACAGCTT	20	865	60.10	6364–7228 (864)
HPV16-L1 fragment 3 (R)	GCACATACAAGCACATACAAACA	23		58.70	

Abbreviations: F, represents forward; R, reverse; bp, base pair; Tm, melting temperature.

**Table 4:** In-house target-specific primers for HPV18 E6 and E7 and the multiple primer sets for L1.

Primer		Length, Bases	Product Size, bp	Tm, °C	Gene Location (Sequence Length)
HPV18-E6 (F)	GGGACCGAAAACGGTGTAT	19	611	59.66	55–665 (610)
HPV18-E6 (R)	GAAGGTCAACCGGAATTTCA	20		59.91	
Sequence		Length, Bases	Product Size, bp	Tm, °C	Gene Location
HPV18-E7 (F)	CTCCAACGACGCAGAGAAAC	20	495	59.22	552–1064 (512)
HPV18-E7 (R)	ACCCTGTGTCTGTTGCATTT	20		57.93	
Sequence		Length, Bases	Product Size, bp	Tm, °C	Gene Location
HPV18-L1 fragment 1 (F)	CGTYCTACTACCTCCTYTGCA	21	700	57.41	5333–6032 (699)
HPV18-L1 fragment 1 (R)	AGAAACATTAGACGTRGCGG	20		58.28	
Sequence		Length, Bases	Product Size, bp	Tm, °C	Gene Location
HPV18-L1 fragment 2 (F)	CAACGTTTAGTGTGGGCCTG	20	698	59.41	5898–6595 (697)
HPV18-L1 fragment 2 (R)	TGATTATGCCAGCARAYACCA	21		58.48	
Sequence		Length, Bases	Product Size, bp	Tm, °C	Gene Location
HPV18-L1 fragment 3 (F)	CCAAGTGGCTCTATTGTTACCTC	23	696	58.81	6532–7136 (604)
HPV18-L1 fragment 3 (R)	ACACARGACATACAAACACAACA	23		58.73	

Abbreviations: F, represents forward; R, reverse; bp, base pair; Tm, melting temperature.

**Table 5:** HPV16 and HPV18 sublineage references for phylogenetic tree construction.

HPV16 Sublineage	Accession Number	Database	HPV18 Sublineage	Accession Number	Database
A1	HQ644280.1	NCBI	A1	AY262282.1	NCBI and PaVE
	HQ644282.1				
	HQ644283.1				
	HQ644268.1				
	NC_001526.4				
A2	KY549170.1	NCBI	A2	EF202146.1	NCBI and PaVE
	AF536179.1	NCBI and PaVE			
A3	AB889494.1	NCBI	A3	EF202147.1	NCBI and PaVE
	HQ644236.1	NCBI and PaVE			
A4	HQ644248.1	NCBI	A4	EF202150.1	NCBI
	AF534061.1	NCBI and PaVE	A5	GQ180787.1	NCBI and PaVE
B1	HQ644290.1	NCBI	B1	EF202155.1	NCBI and PaVE
	HQ644293.1	NCBI			
	AF536180.1	NCBI and PaVE			
B2	KU053915.1	NCBI	B2	KC470225.1	NCBI and PaVE
	HQ644298.1	NCBI and PaVE	B3	EF202152.1	NCBI and PaVE
C	HQ644249.1	NCBI	C	KC470229.1	NCBI and PaVE
	KY549231.1	NCBI		KC470229.1	
	AF472509.1	NCBI and PaVE			
D1	HQ644257.1	NCBI and PaVE	-	-	-
D2	HQ644279.1	NCBI and PaVE	-	-	-
D3	AF402678.1	NCBI and PaVE	-	-	-
	HQ644255.1	NCBI	-	-	-

Abbreviations: PaVE represents PapillomaVirus Episteme; NCBI, National Center for Biotechnology Information.

**Table 6:** Summary of the demographic and clinical characteristics of all our study participants.

	Human Papillomavirus Type 16 Total Sample Size = 54		Human Papillomavirus Type 18 Total Sample Size = 6	
	n (%)	*χ*^2^/(*p*-Value)	n (%)	*χ*^2^/(*p*-Value)
Marital status		36/(*p* < 0.0001) *		NA
Divorced	3 (5.6)		0	
Married	38 (70.3)		6 (100)	
Widowed	13 (24.1)		0	
Age mean (SD)	53.9 (12.9)		48 (8.9)	
Minimum	30		37	
Maximum	82		60	
Previously diagnosed with cervical cancer		57/(*p* < 0.0001) *		NA
Yes	44 (81.5)		6 (100)	
No	8 (14.8)		0	
Unknown	2 (3.7)		0	
Histology results		27.1/(*p* < 0.0001) *		NA
CIN 1	1 (2.9)		0	
CIN 2	3 (8.6)		0	
CIN 3	15 (42.9)		0	
Invasive carcinoma	14 (40)		1 (20)	
Cell carcinoma	0		3 (60)	
NIEL	2 (5.7)		1 (20)	
Cytology test		24.7/(*p* < 0.0001) *		NA
HSIL	11 (25.6)		0	
Invasive carcinoma	29 (67.4)		3 (100)	
NIEL	3 (6.9)		0	
SCC grades, for N = 29		8.7/(*p* < 0.0125) *		NA
SCC Grade I	16 (55.2)		1 (25)	
SCC Grade II	10 (34.5)		1 (25)	
SCC Grade III	3 (10.3)		2 (50)	
AC SCC, N = 29		NA		NA
Adenocarcinoma	2 (6.9)		0	
Viral load Mean (SD), viral copies/µL	5074 (12890)		22850 (49144)	

Abbreviations: CIN 1, mild dysplasia; CIN 2, moderate dysplasia; CIN 3 severe dysplasia; NIEL, negative for intraepithelial lesion; HSIL, high-grade squamous intraepithelial lesion; SCC, squamous cell carcinoma; AC, Adenocarcinoma; NA, not applicable. * Indicates statistical significance (*p* < 0.05). NA, not applicable for statistical testing due to the poor sample size.

**Table 7:** HPV16 gene variants significantly associated with higher viral loads.

Gene	Nucleic Acid/Amino Acid Mutation	Viral Load, Mean (SD), Viral Copies/µL)		Wilcoxon Rank-Sum Test *p*-Value
		Present (Mutation)	Absent (Wild Type)	
E6	145 G -> T	7770.8 (16,798.7)	2026.4 (4854.8)	(0.017) *
	145 G-> T/Q14H	5259.7 (10,039.4)	4992.7 (14103.9)	(0.035) *
	286 T -> A	7510 (16,528.1)	2085.4 (4960.6)	(0.0162) *
	289 A -> G	7510 (16,528.1)	2085.4 (4960.6)	(0.0162) *
	335 C -> T/H78Y	7510 (16,528.1)	2085.4 (4960.6)	(0.0162) *
E7	789 T -> C	7509.7 (16,528)	2085.8 (4960.4)	(0.0174) *
	795 T -> G	7509.7 (16,528)	2085.5 (4960.4)	(0.0174) *
L1	5698 G -> A	7242.1 (16,281)	2184.3 (5060.8)	(0.0448) *
	5864 C -> T/H76Y	7242.1 (16,281)	2184.3 (5060.8)	(0.0448) *
	5911 T -> C	7242.1 (16,281)	2184.3 (5060.8)	(0.0448) *
	6434 A -> G/T266A	5754.3 (14,149.7)	2423.1 (5574.9)	(0.0449) *
	6559 C -> T	7389.1 (17,003.8)	2663.4 (5761.8)	(0.0224) *
	6695 A -> C/T353P	14,596.3 (22,832)	1986.3 (4715.8)	(0.0004) *
	6721 G -> A	14,596.3 (22,832)	1986.3 (4715.8)	(0.0004) *
	6854 C -> T	14,596.3 (22,832)	1986.3 (4715.8)	(0.0004) *
	6865 C -> T	14,596.3 (22,832)	1986.3 (4715.8)	(0.0004) *
	6970 C -> T	15,682.7 (23,619)	2003.7 (4625)	(0.0007) *
	6994 G -> A	17,206 (24,321.1)	1963.8 (4598)	(0.0003) *
	7060 G -> T/L474F	17,206 (24,321.1)	1963.8 (4598)	(0.0003) *

* Indicates statistical significance (*p* < 0.05).

## Data Availability

The data and codes used in this study are available upon request.

## Supplementary Materials

The following supporting information can be downloaded at: https://www.mdpi.com/article/10.3390/v15010109/s1. Table S1: Mutations detected in the E6, E7, and L1 genes of HPV16 in the study population. Table S2. Cervical Cancer association with HPV16 E6 mutations observed in the Saudi population. Table S3. Cervical Cancer association with HPV16 E7 mutations observed in the Saudi population. Table S4. Cervical Cancer association with HPV16 L1 mutations observed in the Saudi population. Table S5. HPV 16 E6 mutations distributed by histology, cytology and SCC grades. Table S6. HPV 16 E7 mutations distributed by histology, cytology and SCC grades. Table S7. HPV 16 L1 mutations distributed by histology, cytology and SCC grades.
