# Supplementary material for: Genetic Variability in the E6, E7, and L1 Genes of Human Papillomavirus Types 16 and 18 among Women in Saudi Arabia
Source: Viruses. 2022 Dec 30;15(1):109. doi: 10.3390/v15010109 (PMC9862970; doi:10.3390/v15010109)
Supplement: Supplementary file 1 [file viruses-15-00109-s001.zip › viruses-2021230-supplementary.pdf]

Table S1. Mutations detected in HPV16 E6, E7, and L1 genes in the study population.

Gene	Nucleic Acid / Amino Acid Mutations	Freq (%)	
		Mutation (A0/A1)	Wild Type (A1/A1)
E6	109 T -> C	5(10.6)	42(89.4)
	132 G -> T / R10I	4(8.5)	43(91.5)
	143 C -> G	8(17)	39(83)
	145 G -> T	26(55.3)	21(44.7)
	143 C -> G and 145 G-> T / Q14D	8(17)	39(83)
	145 G-> T / Q14H	15(31.9)	32(68.1)
	173 C -> A / H24N	1(2.1)	46(97.9)
	176 G -> T / D25Y	1(2.1)	46(97.1)
	178 T -> G / D25E	2(4.3)	45(95.7)
	183 T -> G / I27R	1(2.1)	46(97.5)
	195 T -> C / V31A	1(2.1)	46(97.5)
	285 C -> G / A61G	1(2.1)	46(97.5)
	286 T -> A	27(57.4)	20(42.6)
	289 A -> G	27(57.4)	20(42.6)
	295 T -> G / D64E	1(2.1)	46(97.5)
	335 C -> T / H78Y	27(57.4)	20(42.6)
	350 T -> G / L83V	27(57.4)	20(42.6)
	403 A -> G	5(10.6)	42(89.4)
	533 A -> G	5(10.6)	42(89.4)
E7	645 A ->C / L28F	1(1.92)	51(98.08)
	647 A ->G / N29S	8(15.38)	44(84.62)
	666 G -> A	1(1.92)	51(98.08)
	732 T->C	5(9.62)	47(90.38)
	735 T -> C	11 (21.15)	41(78.85)
	789 T -> C	29(55.77)	23(44.23)
	795 T -> G	29(55.77)	23(44.23)
	795 T -> C	1(1.92)	51(98.08)
	822 A -> G	1(1.92)	51(98.08)
	843 T -> C	1(1.92)	51(98.08)
	846 T -> C	2(3.85)	50(96.15)
L1	5659 T -> C	1(1.96)	50(98.04)
	5698 G -> A	29 (56.86)	22(43.14)
	5791 T -> C	2(3.92)	49(96.08)
	5796 A -> C / K53T	1(1.96)	50(98.04)
	5797 A -> C / K53N	1(1.96)	50(98.04)

	**5797 A -> G**	1(1.96)	50(98.04)
	**5864 C -> T /H76Y**	29 (56.86)	22(43.14)
	**5911 T -> C**	29 (56.86)	22(43.14)
	**5962 T -> C**	1(1.96)	50(98.04)
	**6001 T -> G**	1(1.96)	50(98.04)
	**6016 G -> A**	1(1.96)	50(98.04)
	**6025 A -> C**	1(1.96)	50(98.04)
	**6061 G -> A**	1(1.96)	50(98.04)
	**6070 A -> G**	1(1.96)	50(98.04)
	**6165 C -> A / T176N**	28(54.90)	23(45.10)
	**6180 A -> G / N181S**	3 (5.88)	48 (94.12)
	**6180 A -> C/ N181T**	5 (9.80)	46 (90.20)
	**6218 G -> A/ V194I**	1(1.96)	50(98.04)
	**6247 T -> C**	28(54.90)	23(45.10)
	**6316 A -> G**	24 (47.06)	27 (52.94)
	**6391 A -> G**	1(1.96)	50(98.04)
	**6434 A -> G / T266A**	41 (80.39)	10 (19.61)
	**6482 T -> C / S282P**	8 (15.69)	43 (84.31)
	**6559 C -> T**	26 (50.98)	25 (49.02)
	**6568 T -> A**	1(1.96)	50(98.04)
	**6583 A -> C**	2(3.92)	49(96.08)
	**6689 T -> G / S351A**	1(1.96)	50(98.04)
	**6695 A -> C / T353P**	12 (23.53)	39 (76.47)
	**6721 G -> A**	12 (23.53)	39 (76.47)
	**6732 G -> C / R365P**	1(1.96)	50(98.04)
	**6803 A-> T / T389S**	1(1.96)	50(98.04)
	**6824 T -> C/ S396P**	1(1.96)	50(98.04)
	**6826 C -> T**	1(1.96)	50(98.04)
	**6854 C -> T**	12 (23.53)	39 (76.47)
	**6865 C -> T**	12 (23.53)	39 (76.47)
	**6868 A -> G**	1(1.96)	50(98.04)
	**6952 A -> G**	1(1.96)	50(98.04)
	**6967 A -> C / K443N**	1(1.96)	50(98.04)
	**6970 C -> T**	11(21.57)	40 (78.43)
	**6994 G -> A**	10 (19.61)	41 (80.39)
	**7060 G -> A**	3 (5.88)	48 (94.12)
	**7060 G ->T / L474F**	10 (19.61)	41 (80.39)

Table S2. Cervical Cancer association with HPV16 E6 mutations observed in the Saudi population.

Nucleic Acid / Amino Acid Mutations (Positive)	Freq (%)		OR (95%, CI)	*Fisher exact test, P-Value*
	Cervical Cancer	Non-cervical cancer		
**109 T -> C (N=5)**	4(80)	1(20)	0.7(0.06-7.22)	1
**132 G -> T / R10I (N=4)**	3(75)	1(25)	0.5(0.04-5.65)	0.49
**143 C -> G (N=8)**	2(25)	6(75)	0.47(0.07-2.91)	0.59
**145 G -> T (N=26)**	2(7.6)	24(92.4)	3.6(0.65-19.8)	0.24
**143 C -> G and 145 G-> T / Q14D**	6(75)	2(25)	0.47(0.07-2.91)	0.59
**145 G-> T / Q14H (N=15)**	15(100)	0(0)	0.78(0.66-0.92)	0.087
**173 C -> A / H24N (N=1)**	1(100)	0(0)	0.84(0.74-0.94)	1
**176 G -> T / D25Y0(N=1)**	1(100)	0(0)	0.84(0.74-0.94)	1
**178 T -> G / D25E (N=2)**	1(50)	1(50)	0.16(0.01-2.9)	0.29
**183 T -> G / I27R (N=1)**	1(100)	0(0)	0.84(0.74-0.94)	1
**195 T -> C / V31A (N=1)**	1(100)	0(0)	0.84(0.74-0.94)	1
**285 C -> G / A61G (N=1)**	1(100)	0(0)	0.84(0.74-0.94)	1
**286 T -> A (N=27)**	25(92.6)	2(7.4)	3.94(0.71-21.8)	0.134
**289 A -> G (N=27)**	25(92.6)	2(7.4)	3.94(0.71-21.8)	0.134
**295 T -> G / D64E (N=1)**	1(100)	0(0)	0.84(0.74-0.94)	1
**335 C -> T / H78Y (N=27)**	25(92.6)	2(7.4)	3.94(0.71-21.8)	0.134
**350 T -> G / L83V (N=27)**	26(96.3)	1(3.7)	10.1(1.1-89.4)	0.022*
**403 A -> G (N=5)**	4(80)	1(20)	0.7(0.07-7.2)	1
**533 A -> G (N=5)**	5(100)	0(0)	0.82(.72-0.94)	1

Table S3. Cervical Cancer association with HPV16 E7 mutations observed in the Saudi population.

**Nucleic Acid / Amino Acid Mutations (Positive)**	**Freq (%)**		**OR (95%, CI)**	***Fisher exact test P-Value***
	**Cervical Cancer**	**Noncervical cancer**		
**645 A ->C / L28F (N=1)**	0(0)	1(100)	0.14(0.07-0.27)	0.1538
**647 A ->G / N29S (N=8)**	5(62.5)	3(37.5)	0.21(0.04-1.18)	0.094
**666 G -> A (N=1)**	1(100)	0(0)	0.84(0.74-0.94)	1
**732 T->C (N=5)**	5(100)	0(0)	0.84(0.74-0.94)	1
**735 T -> C (N=11)**	11(100)	0(0)	0.80(0.69—0.93)	0.18
**789 T -> C (N=29)**	27(93.1)	2(6.9)	4.8(0.86-26.4)	0.12
**795 T -> G (N=29)**	27(93.1)	2(5.9)	4.8(0.86-26.4)	0.12
**795 T -> C (N=1)**	1(100)	0(0)	0.84(0.74-0.94)	1
**822 A -> G (N=1)**	1(100)	0(0)	0.84(0.74-0.94)	1
**843 T -> C (N=1)**	1(100)	0(0)	0.84(0.74-0.94)	1
**846 T -> C (N=2)**	1(50)	1 (50)	0.16(0.01-2.91)	0.29

Table S4. Cervical Cancer association with HPV16 L1 mutations observed in the Saudi population.

**Nucleic Acid / Amino Acid Mutations (Positive)**	**Freq (%)**		**OR (95%, CI)**	***Fisher exact test P-Value***
	**Cervical Cancer**	**Noncervical cancer**		
**5659 T -> C (N=1)**	1(100)	0(0)	0.84(0.74-0.94)	1
**5698 G -> A (N=29)**	27(93.1)	2(6.9)	4.8(0.86-26.4)	0.12
**5791 T -> C (N=2)**	2(100)	0(0)	0.84(0.74-0.94)	1
**5796 A -> C / K53T (N=1)**	1(100)	0(0)	0.84(0.74-0.94)	1
**5797 A -> C / K53N (N=1)**	1(100)	0(0)	0.84(0.74-0.94)	1
**5797 A -> G (N=1)**	1(100)	0(0)	0.84(0.74-0.94)	1
**5864 C -> T /H76Y (N=29)**	27(93.1)	2(6.9)	4.8(0.86-26.4)	0.12
**5911 T -> C (N=29)**	27(93.1)	2(6.9)	4.8(0.86-26.4)	0.12
**5962 T -> C (N=1)**	1(100)	0(0)	0.84(0.74-0.94)	1
**6001 T -> G (N=1)**	1(100)	0(0)	0.84(0.74-0.94)	1
**6016 G -> A (N=1)**	1(100)	0(0)	0.84(0.74-0.94)	1
**6025 A -> C (N=1)**	1(100)	0(0)	0.84(0.74-0.94)	1
**6061 G -> A (N=1)**	1(100)	0(0)	0.84(0.74-0.94)	1
**6070 A -> G (N=1)**	1(100)	0(0)	0.84(0.74-0.94)	1
**6165 C -> A / T176N (N=28)**	26(92.9)	2(7.1)	4.3(0.78-23.9)	0.123
**6180 A -> G / N181S (N=3)**	2(66.7)	1(33.3)	0.33(0.026-4.2)	0.40
**6180 A -> C/ N181T (N=5)**	5(100)	0(0)	0.82(0.72-0.94)	1
**6218 G -> A/ V194I (N=1, UNK)**	NA	NA	NA	NA
**6247 T -> C (N=28)**	26(92.9)	2(7.14)	4.3(0.78-23.9)	0.123
**6316 A -> G (N=24)**	22(91.7)	2(8.3)	3(0.54-16.5)	0.26
**6391 A -> G (N=1)**	1(100)	0(0)	0.84(0.74-0.94)	1
**6434 A -> G / T266A (N=40)**	36(90)	4(10)	4.5(0.92-21.9)	0.072

**6482 T -> C / S282P (N=8)**	6(75)	2(25)	0.47(0.07-2.9)	0.59
**6559 C -> T (N=26)**	24(92.3)	2(7.7)	3.6(0.65-19.8)	0.24
**6568 T -> A (N=1)**	1(100)	0(0)	0.84(0.74-0.95)	1
**6583 A -> C (N=2)**	2(100)	0(0)	0.84(0.74-0.95)	1
**6689 T -> G / S351A (N=1)**	1(100)	0(0)	0.84(0.74-0.95)	1
**6695 A -> C / T353P (N=12)**	11(92.7)	1(8.3)	2.3(0.26-21.1)	0.66
**6721 G -> A (N=12)**	11(92.7)	1(8.3)	2.3(0.26-21.1)	0.66
**6732 G -> C / R365P (N=1)**	1(100)	0(0)	0.84(0.74-0.95)	1
**6803 A-> T / T389S (N=1)**	1(100)	0(0)	0.84(0.74-0.95)	1
**6824 T -> C/ S396P (N=1)**	1(100)	0(0)	0.84(0.74-0.95)	1
**6826 C -> T (N=1)**	1(100)	0(0)	0.84(0.74-0.95)	1
**6854 C -> T (N=12)**	11(92.7)	1(8.3)	2.3(0.26-21.1)	0.66
**6865 C -> T (N=12)**	11(92.7)	1(8.3)	2.3(0.26-21.1)	0.66
**6868 A -> G (N=1)**	1(100)	0(0)	0.84(0.74-0.95)	1
**6952 A -> G (N=1)**	1(100)	0(0)	0.84(0.74-0.95)	1
**6967 A -> C / K443N (N=1)**	1(100)	0(0)	0.84(0.74-0.95)	1
**6970 C -> T (N=11)**	10(90.9)	1(9.1)	2.1(0.25-18.8)	1
**6994 G -> A (N=10)**	9(90)	1(10)	1.8(0.19-16.6)	1
**7060 G -> A (N=3)**	2(66.7)	1(33.3)	0.3(0.02-4.18)	0.40
**7060 G ->T / L474F (N=10)**	9(90)	1(10)	1.8(0.19-16.6)	1

Table S5. HPV 16 E6 mutations distributed by histology, cytology and SCC grades.

	A) Histology (AA0/AA), Mutation N/ Wild Type N					
**E6 Mutations**	**CIN 1**	**CIN 2**	**CIN 3**	**Invasive Carcinoma**	**NILE**	**X^2, P value**
**109 T -> C**	0/1	0/3	2/13	1/13	0/2	1.03(0.91)
**132 G -> T / R10I**	0/1	0/3	2/13	0/14	0/2	2.82(0.58)
**143 C -> G**	0/1	0/3	3/12	1/13	0/2	2.1(0.71)
**145 G -> T**	0/1	1/2	8/7	8/6	0/2	3.7(0.45)
**143 C -> G and 145 G-> T / Q14D**	0/1	0/3	3/12	1/13	0/2	2.1(0.71)
**145 G-> T / Q14H**	0/1	1/2	2/13	7/7	0/2	6.1(0.19)
**173 C -> A / H24N**	0/1	0/3	1/14	0/14	0/2	1.37(0.84)
**176 G -> T / D25Y**	0/1	0/3	0/15	0/14	0/2	NA
**178 T -> G / D25E**	0/1	0/3	0/15	0/14	0/2	NA
**183 T -> G / I27R**	0/1	0/3	1/14	0/14	0/2	1.37(0.84)
**195 T -> C / V31A**	0/1	0/3	0/15	0/14	0/2	NA
**285 C -> G / A61G**	0/1	0/3	1/14	0/14	0/2	1.37(0.84)
**286 T -> A**	0/1	1/2	8/7	9/5	0/2	4.5(0.34)
**289 A -> G**	0/1	1/2	8/7	9/5	0/2	4.5(0.34)
**295 T -> G / D64E**	0/1	0/3	0/15`	1/13	0/2	1.5(0.82)
**335 C -> T / H78Y**	0/1	1/2	8/7	9/5	0/2	4.5(0.34)
**350 T -> G / L83V**	0/1	1/2	9/6	10/4	0/2	5.9(0.21)
**403 A -> G**	0/1	0/3	2/13	1/13	0/2	1.03(0.90)
**533 A -> G**	0/1	0/3	2/13	2/12	0/2	0.94(0.91)

	B) Cytology Test (AA0/AA), Mutation N/ Wild Type N			
**E6 Mutations**	**HSIL**	**Invasive Carcinoma**	**NILE**	**X^2, P value**
**109 T -> C**	2/9	1/28	0/3	2.9(0.23)
**132 G -> T / R10I**	2/9	1/28	0/3	6.1(0.047) *

**143 C -> G**	3/8	3/26	0/3	2.4(0.29)
**145 G -> T**	5/6	16/13	0/3	3.4(0.18)
**143 C -> G and 145 G-> T / Q14D**	3/8	3/26	0/3	2.43(0.30)
**145 G-> T / Q14H**	1/10	12/17	0/3	5.3(0.069)
**173 C -> A / H24N**	1/10	0/29	0/3	2.9(0.22)
**176 G -> T / D25Y**	0/11	1/28	0/3	0.49(0.78)
**178 T -> G / D25E**	1/10	1/28	0/3	1.72(0.69)
**183 T -> G / I27R**	1/10	0/29	0/3	2.9(0.22)
**195 T -> C / V31A**	0/11	1/28	0/3	0.49(0.78)
**285 C -> G / A61G**	0/11	0/29	0/3	NA
**286 T -> A**	5/6	17/12	0/3	3.9(0.14)
**289 A -> G**	5/6	17/12	0/3	3.9(0.14)
**295 T -> G / D64E**	0/11	1/28	0/3	0.49(0.78)
**335 C -> T / H78Y**	6/5	17/12	0/3	3.9(0.14)
**350 T -> G / L83V**	4/7	18/11	0/3	5.5(0.064)
**403 A -> G**	2/9	1/28	0/3	2.9(0.23)
**533 A -> G**	2/9	3/26	0/3	0.90(0.63)
**C) SCC Grades for mutations with intensive carcinoma**				
**E6 Mutations**	**SCC**	**SCC Grade II**	**SCC Grade III**	**X^2, P value**
**109 T -> C**	0/16	1/9	0/3	1.9(0.37)
**132 G -> T / R10I**	0/16	0/10	0/3	NA
**143 C -> G**	1/15	1/9	1/2	1.9(0.37)
**145 G -> T**	7/9	7/3	3/0	4.1(0.13)
**143 C -> G and 145 G-> T / Q14D**	1/15	1/9	1/2	1.9(0.36)
**145 G-> T / Q14H**	5/11	6/4	2/1	2.7(0.25)

**173 C -> A / H24N**	0/16	0/10	0/3	NA
**176 G -> T / D25Y**	0/16	0/10	0/3	NA
**178 T -> G / D25E**	0/16	0/10	0/3	NA
**183 T -> G / I27R**	0/16	0/10	0/3	NA
**195 T -> C / V31A**	1/15	0/10	0/3	0.84(0.65)
**285 C -> G / A61G**	0/16	0/10	0/3	NA
**286 T -> A**	7/9	8/2	3/0	5.5(0.064)
**289 A -> G**	7/9	8/2	3/0	5.5(0.064)
**295 T -> G / D64E**	0/16	1/9	0/3	1.9(0.37)
**335 C -> T / H78Y**	9/7	8/2	3/0	5.5(0.065)
**350 T -> G / L83V**	9/7	7/3	2/1	0.52(0.77)
**403 A -> G**	0/16	1/9	0/3	1.9(0.37)
**533 A -> G**	2/14	2/8	0/3	0.82(0.66)

Table S6. HPV 16 E7 mutations distributed by histology, cytology and SCC grades.

	**A) Histology (AA0/AA), Mutation N/ Wild Type N**					
**E7 Mutations**	**CIN 1**	**CIN 2**	**CIN 3**	**Invasive Carcinoma**	**NILE**	**X^2, P value**
**645 A ->C / L28F**	0/1	0/3	0/15	0/14	0/2	35(<0.0001) *
**647 A ->G / N29S**	0/1	0/3	3/12	1/13	0/2	2.1(0.71)
**666 G -> A**	0/1	0/3	0/15	1/13	0/2	1.54(0.81)
**732 T->C**	0/1	0/3	2/13	2/12	0/2	0.94(0.92)
**735 T -> C**	0/1	1/2	2/13	3/11	0/2	1.50(0.82)
**789 T -> C**	0/1	2/1	8/7	9/5	0/2	4.3(0.37)
**795 T -> G**	0/1	2/1	8/7	9/5	0/2	4.3(0.37)
**795 T -> C**	0/1	1/2	0/15	0/14	0/2	10.9(0.027) *
**822 A -> G**	0/1	0/3	0/15	1/13	0/2	1.5(0.82)

**843 T -> C**	0/1	0/3	0/15	0/14	0/2	NA
**846 T -> C**	0/1	0/3	0/15	0/14	0/2	NA

	**B) Cytology Test (AA0/AA), Mutation N/ Wild Type N**			
**E7 Mutations**	**HSIL**	**Invasive Carcinoma**	**NIEL**	**X^2, P value**
**645 A ->C / L28F**	0/11	0/29	0/3	NA
**647 A ->G / N29S**	4/7	3/26	0/3	4.6(0.10)
**666 G -> A**	0/11	1/28	0/3	0.49(0.78)
**732 T->C**	2/9	3/26	0/3	0.90(0.64)
**735 T -> C**	0/11	9/20	0/3	5.5(0.064)
**789 T -> C**	6/5	19/10	0/3	4.9(0.088)
**795 T -> G**	6/5	19/10	0/3	4.9(0.088)
**795 T -> C**	0/11	1/28	0/3	0.5(0.78)
**822 A -> G**	0/11	0/29	0/3	NA
**843 T -> C**	0/11	1/28	0/3	0.50(0.78)
**846 T -> C**	1/10	1/28	0/3	0.72(0.69)

	**SCC Classification**			
**E7 Mutations**	**SCC**	**SCC Grade I**	**SCC Grade II**	**X^2, P value**
**645 A ->C / L28F**	0/16	0/10	0/3	NA
**647 A ->G / N29S**	1/15	1/9	0/3	0.38(0.82)
**666 G -> A**	0/16	1/9	0/3	1.96(0.37)
**732 T->C**	2/14	2/8	0/3	0.82(0.67)
**735 T -> C**	5/11	3/7	1/2	0.01(0.99)
**789 T -> C**	9/7	8/2	3/0	3.1(0.20)
**795 T -> G**	9/7	8/2	3/0	3.1(0.20)
**795 T -> C**	1/15	0/10	0/3	0.84(0.65)
**822 A -> G**	0/16	0/10	0/3	NA
**843 T -> C**	0/16	0/10	0/3	NA

**846 T -> C**	0/16	0/10	0/3	NA

Table S7. HPV 16 L1 mutations distributed by histology, cytology and SCC grades.

	A) Histology (AA0/AA), Mutation N/ Wild Type N					
**L1 Mutations**	**CIN 1**	**CIN 2**	**CIN 3**	**Invasive Carcinoma**	**NILE**	**X^2, P value**
**5659 T -> C**	0/1	0/3	0/15	1/13	0/2	1.54(0.82)
**5698 G -> A**	0/1	2/1	5/10	9/5	0/2	5.5(0.23)
**5791 T -> C**	0/1	0/3	1/14	1/13	0/2	0.44(0.97)
**5796 A -> C / K53T**	0/1	0/3	0/15	0/14	0/2	NA
**5797 A -> C / K53N**	0/1	0/3	0/15	0/14	0/2	NA
**5797 A -> G**	0/1	0/3	1/14	0/14	0/2	1.4(0.85)
**5864 C -> T /H76Y**	0/1	1/2	10/5	9/5	0/2	5.5(0.23)
**5911 T -> C**	0/1	1/2	10/5	9/5	0/2	5.5(0.23)
**5962 T -> C**	0/1	0/3	0/15	0/14	0/2	NA
**6001 T -> G**	0/1	0/3	0/15	1/13	0/2	1.5(0.82)
**6016 G -> A**	0/1	0/3	0/15	1/13	0/2	1.5(0.82)
**6025 A -> C**	0/1	0/3	1/14	0/14	0/2	1.4(0.85)
**6061 G -> A**	0/1	0/3	0/15	1/13	0/2	1.5(0.82)
**6070 A -> G**	0/1	0/3	0/15	0/14	0/2	NA
**6165 C -> A / T176N**	0/1	1/2	10/5	9/5	0/2	5.5(0.23)
**6180 A -> G / N181S**	0/1	0/3	1/14	0/14	0/2	1.4(0.85)
**6180 A -> C/ N181T**	0/1	0/3	2/13	2/12	0/2	0.94(0.92)
**6218 G -> A/ V194I**	0/1	0/3	0/15	0/14	0/2	NA
**6247 T -> C**	0/1	1/2	10/5	9/5	0/2	5.5(0.23)
**6316 A -> G**	0/1	½	9/6	7/7	0/2	3.9(0.41)
**6391 A -> G**	0/1	0/3	0/15	1/13	1/13	1.5(0.82)

**6434 A -> G / T266A**	0/1	1/2	13/2	13/1	1/1	11.1(0.025) *
**6482 T -> C / S282P**	0/1	0/3	4/11	1/13	0/2	3.5(0.49)
**6559 C -> T**	0/1	1/2	7/8	9/5	0/2	4.5(0.34)
**6568 T -> A**	0/1	0/3	0/15	1/13	0/2	1.54(0.81)
**6583 A -> C**	0/1	0/3	2/13	0/14	0/2	2.8(0.59)
**6689 T -> G / S351A**	0/1	0/3	0/15	1/13	0/2	1.54(0.82)
**6695 A -> C / T353P**	0/1	1/2	1/14	6/8	0/2	6.5(0.16)
**6721 G -> A**	0/1	1/2	1/14	6/8	0/2	6.5(0.16)
**6732 G -> C / R365P**	0/1	0/3	0/15	0/14	0/2	NA
**6803 A-> T / T389S**	0/1	0/3	0/15	1/13	0/2	1.5(0.82)
**6824 T -> C/ S396P**	0/1	0/3	0/15	1/13	0/2	1.5(0.82)
**6826 C -> T**	0/1	0/3	0/15	1/13	0/2	1.5(0.82)
**6854 C -> T**	0/1	1/2	1/14	6/8	0/2	6.5(0.16)
**6865 C -> T**	0/1	1/2	1/14	6/8	0/2	6.5(0.16)
**6868 A -> G**	0/1	0/3	1/14	0/14	0/2	1.4(0.85)
**6952 A -> G**	0/1	0/3	1/14	0/14	0/2	1.4(0.85)
**6967 A -> C / K443N**	0/1	0/3	1/14	0/14	0/2	1.4(0.85)
**6970 C -> T**	0/1	0/3	1/14	6/8	0/2	7.7(0.10)
**6994 G -> A**	0/1	0/3	0/15	6/8	0/2	10.9(0.028) *
**7060 G -> A**	0/1	0/3	1/14	1/13	0/2	0.44(0.97)
**7060 G ->T / L474F**	0/1	0/3	0/15	6/8	0/2	10.9(0.028) *
	**B) Cytology Test (AA0/AA), Mutation N/ Wild Type N**					
**L1 Mutations**	**HSIL**	**Invasive Carcinoma**		**NIEL**	**X^2, P value**	
**5659 T -> C**	0/11	1/28		0/3	0.49(0.78)	
**5698 G -> A**	5/6	18/11		0/3	4.5(0.100)	

**5791 T -> C**	1/10	1/28	0/3	0.72(0.69)
**5796 A -> C / K53T**	0/11	1/28	0/3	0.49(0.78)
**5797 A -> C / K53N**	0/11	1/28	0/3	0.49(0.78)
**5797 A -> G**	0/11	0/29	0/3	NA
**5864 C -> T /H76Y**	5/6	18/11	0/3	4.6(0.10)
**5911 T -> C**	5/6	18/11	0/3	4.6(0.10)
**5962 T -> C**	0/11	1/28	0/3	0.49(0.78)
**6001 T -> G**	0/11	1/28	0/3	0.49(0.78)
**6016 G -> A**	0/11	1/28	0/3	0.49(0.78)
**6025 A -> C**	1/10	0/29	0/3	2.9(0.22)
**6061 G -> A**	0/11	1/28	0/3	0.49(0.78)
**6070 A -> G**	0/11	1/28	0/3	0.49(0.78)
**6165 C -> A / T176N**	5/6	18/11	0/3	4.6(0.100)
**6180 A -> G / N181S**	1/10	1/28	0/3	0.30(0.86)
**6180 A -> C/ N181T**	1/0	3/26	0/3	0.34(0.84)
**6218 G -> A/ V194I**	0/11	0/29	1/2	13.7(0.001) *
**6247 T -> C**	5/6	18/11	0/3	4.6(0.100)
**6316 A -> G**	4/7	15/14	0/3	3.3(0.19)
**6391 A -> G**	0/11	0/29	0/3	NA
**6434 A -> G / T266A**	7/4	25/4	2/1	2.8(0.25)
**6482 T -> C / S282P**	3/8	4/25	0/3	1.7(0.42)
**6559 C -> T**	5/6	18/11	0/3	4.5(0.100)
**6568 T -> A**	0/11	0/29	0/3	0.49(0.78)
**6583 A -> C**	0/11	1/28	0/3	0.49(0.78)
**6689 T -> G / S351A**	0/11	1/28	0/3	0.49(0.78)
**6695 A -> C / T353P**	2/9	8/21	0/3	1.37(0.50)
**6721 G -> A**	2/9	8/21	0/3	1.37(0.50)

**6732 G -> C / R365P**	1/10	0/29	0/3	2.9(0.22)
**6803 A-> T / T389S**	1/10	0/29	0/3	2.9(0.22)
**6824 T -> C/ S396P**	1/10	0/29	0/3	2.9(0.22)
**6826 C -> T**	0/11	1/28	0/3	0.49(0.78)
**6854 C -> T**	2/9	8/21	0/3	1.37(0.50)
**6865 C -> T**	2/9	8/21	0/3	1.37(0.50)
**6868 A -> G**	0/11	1/28	0/3	0.49(0.78)
**6952 A -> G**	0/11	1/28	0/3	0.49(0.78)
**6967 A -> C / K443N**	0/11	1/28	0/3	0.49(0.78)
**6970 C -> T**	2/9	8/21	0/3	1.37(0.50)
**6994 G -> A**	2/9	8/21	0/3	1.37(0.50)
**7060 G -> A**	1/10	2/27	0/3	0.36(0.86)
**7060 G ->T / L474F**	2/9	8/21	0/3	1.37(0.50)

**C) SCC Classifications (AA0/AA), Mutation N/ Wild Type N**				
**L1 Mutations**	**SCC**	**SCC Grade II**	**SCC Grade III**	**P Value**
**5659 T -> C**	0/16	1/9	0/3	1.96(0.37)
**5698 G -> A**	8/8	8/2	0/3	4.2(0.12)
**5791 T -> C**	0/16	1/9	0/3	1.96(0.37)
**5796 A -> C / K53T**	1/15	0/10	0/3	0.84(0.65)
**5797 A -> C / K53N**	1/15	0/10	0/3	0.84(0.65)
**5797 A -> G**	0/16	0/10	0/3	NA
**5864 C -> T /H76Y**	8/8	8/2	3/0	4.2(0.12)
**5911 T -> C**	8/8	8/2	3/0	4.2(0.12)
**5962 T -> C**	0/16	0/10	0/3	NA
**6001 T -> G**	0/16	1/9	0/3	1.96(0.37)
**6016 G -> A**	1/15	0/10	0/3	0.84(0.65)
**6025 A -> C**	0/16	0/10	0/3	NA

**6061 G -> A**	0/16	1/9	0/3	1.96(0.37)
**6070 A -> G**	1/15	0/10	0/3	0.84(0.65)
**6165 C -> A / T176N**	8/8	8/2	3/0	4.2(0.12)
**6180 A -> G / N181S**	1/15	0/10	1/2	4.0(0.13)
**6180 A -> C/ N181T**	2/14	2/8	0/3	0.82(0.66)
**6218 G -> A/ V194I**	0/16	0/10	0/3	NA
**6247 T -> C**	8/8	8/2	3/0	4.2(0.12)
**6316 A -> G**	6/10	6/4	3/0	4.4(0.11)
**6391 A -> G**	0/16	0/10	0/3	NA
**6434 A -> G / T266A**	12/4	9/1	3/0	1.7(0.43)
**6482 T -> C / S282P**	2/14	1/9	1/2	1.1(0.57)
**6559 C -> T**	8/8	8/2	3/0	4.2(0.12)
**6568 T -> A**	0/16	1/9	0/3	1.96(0.37)
**6583 A -> C**	1/15	0/10	0/3	0.84(0.65)
**6689 T -> G / S351A**	0/16	1/9	0/3	1.96(0.37)
**6695 A -> C / T353P**	3/13	3/7	3/0	7.8(0.02) *
**6721 G -> A**	3/13	3/7	3/0	7.8(0.02) *
**6732 G -> C / R365P**	1/15	0/10	0/3	0.84(0.65)
**6803 A-> T / T389S**	1/15	0/10	0/3	0.84(0.65)
**6824 T -> C/ S396P**	0/16	1/9	0/3	1.96(0.37)
**6826 C -> T**	0/16	1/9	0/3	1.96(0.37)
**6854 C -> T**	3/13	3/7	3/0	7.8(0.02) *
**6865 C -> T**	3/13	3/7	3/0	7.8(0.02)
**6868 A -> G**	1/15	0/10	0/3	0.84(0.65)
**6952 A -> G**	1/15	0/10	0/3	0.84(0.65)
**6967 A -> C / K443N**	1/15	0/10	0/3	0.84(0.65)
**6970 C -> T**	3/13	3/7	3/0	7.8(0.02)

**6994 G -> A**	3/13	3/7	3/0	7.8(0.02)
**7060 G -> A**	0/16	1/9	0/3	1.96(0.37)
**7060 G ->T / L474F**	3/13	3/7	3/0	7.8(0.02)
